# The speed of change in climate and land cover is associated with the speed of biodiversity changes in avian assemblages of the United States

**DOI:** 10.1371/journal.pone.0330153

**Published:** 2025-08-29

**Authors:** Marlen Acosta Alamo, Lisa L. Manne

**Affiliations:** 1 Biology Department, College of Staten Island, City University of New York, Staten Island, New York, United States of America; 2 Biology Program, Graduate Center, City University of New York, New York, United States of America; Oklahoma State University, UNITED STATES OF AMERICA

## Abstract

Changes in climate and land use land cover are a widely recognized threat to the stability of natural species assemblages’ composition and biodiversity. Species-specific responses to these changes can result in a rearrangement of the species composition of assemblages, altering the stability, resilience, and functioning of the ecosystems of which these assemblages are a part. We assessed the relationship between the rate of change in avian species richness and assemblage dissimilarity and the rate of change in climatic and land use land cover variables across 30 years in five ecoregions of the United States. Areas where effects of changing land use land cover and/or climate were most strongly felt were high elevations and latitudes. Rates of species replacement and loss were associated with changing environmental factors in opposite directions. Rates of change in biodiversity were more strongly predicted by rates of change in land use land cover than by rates of change in climate. For the species assemblages studied here, rapidly changing climate and/ or land use land cover was more strongly affecting total assemblage dissimilarity patterns than species richness even though species richness has received much more research attention. Trends in multiple biodiversity indices capture multiple levels of action (richness vs. assemblage dissimilarity). A study that integrates these allows us to observe the complex and changing interrelationships between biodiversity and the environment (climate and land cover), and thus, plan effectively for preservation of processes that generate patterns of biodiversity.

## Introduction

Climate change is one of the current drivers of the increasing similarity of species composition among natural species assemblages (i.e., biotic homogenization) [1–5]. Nevertheless, climate change is not new, and studies in fossil avifauna and pollen records show how species assemblages of birds and plants changed around 12,000 years ago, in response to these changes in climate [6,7]. What is alarming, however, is the rapid rates at which climate changes are occurring and their predicted acceleration in the future [8–10]. For example, in the last 200 years, abrupt vegetation changes were significantly higher than at any time in the previous 7,000 years [11]. Moreover, under current best estimates of future climate change, one in six species will face extinction by the end of this century [12].

Land use and land cover (LULC) change is another driver of biotic homogenization linked to population declines and species loss [13,14], which poses threats 3–10 times higher in magnitude than those due to climate change [15]. However, the relative importance of these two environmental factors as drivers of biodiversity loss in terrestrial ecosystems appears to be scale-dependent; changes in LULC have a greater impact on population trends while changes in climate affect species assemblage composition [16]. Nevertheless, the relative importance of these factors may switch over time: meaning that the projected impacts of climate change on vertebrate biodiversity could match or exceed the effect of historical LULC change by the year 2070 [17]. Further, other authors have found that the relative importance of climate change versus LULC change as drivers of change in biodiversity varies across biodiversity dimensions and habitat or ecoregion types [18,19]. This underscores the importance of considering multiple environmental factors in community ecology since threats to species are not discrete, and the majority of assemblages experience a combination of changes in climate and LULC [15].

Changes in climate and LULC are interrelated [20,21] and can interact [22,23]. Their simultaneous effect has been investigated across multiple taxa and biomes [24–32]. Changes in these two drivers could result in no change of the species assemblages, but could conversely lead to either biotic homogenization or heterogenization (for example, [25,31]), potentially impacting the stability and resilience of the ecosystem, as well as its functions and the services it provides [23,33–36]. It is important, however, to also understand how rapidly natural assemblages are responding to different rates of changes in environmental conditions.

Studies comparing climate velocity with species responses have found that whether species’ paces match – or lag behind – climate change is species-specific [37–39]. Just a few studies have considered the combined velocities of climate and LULC change [40,41]. We have previously found that within-ecoregion changes in avian richness and assemblage dissimilarity have occurred rapidly in the last 30 years in the United States [42]. However, the effect of different rates of changes in climate and LULC on the rate of change of biodiversity of current species assemblages, where species may or may not have tracked those changes, has yet to be assessed. Further, the rates of change in climate and LULC may be quite different across ecoregions or biomes [8,41]. For example, in the United States, for year 2050, relatively rapid changes in both climate and LULC are predicted for the Midwest region, while relatively rapid changes in climate but not in LULC are predicted for the Southwest region [41]. Thus, the mechanisms through which avian communities respond to different rates and directions of changes in multiple environmental predictors can also differ across ecoregions at a continental scale. Our study is one of the first to perform a long-term context-dependent analysis of the relationship between the rate of change of biodiversity and the rate of change in the environment with a comparison across ecoregions of the United States.

We can test hypotheses about rates of change in LULC or climate in different ecoregions against rates of change in biodiversity, where biodiversity is defined as species richness or assemblage dissimilarity. Although species richness has received much more research attention, it is important to assess how environmental changes affect biodiversity at different scales of ecological analysis (e.g., comparing environmental effects on species richness and assemblage dissimilarity). Particularly, the components of assemblage dissimilarity, species replacement and species loss or gain [43], could all be important. Including the components of assemblage dissimilarity in the analysis allows for a more nuanced interpretation of the processes behind the biodiversity patterns observed.

Human-induced changes in climatic conditions and the loss or degradation of habitats that accompanies the conversion of natural LULC to human-modified ones, directly impact the suitability of an area for the species typically found there. Changes in habitat suitability can determine the presence (or absence) of individual species in particular locations through different mechanisms. For example, unmet species-specific physiological and/or ecological requirements can result in phenological mismatches [44,45] and/or altered species interactions [44,46] that can ultimately affect a species geographical distribution. When the responses of multiple individual species to changes in habitat suitability are analyzed together across time and space, the impact of these changes on the diversity of avian assemblages emerges.

In this study, we combined spatial and temporal approaches to assess the effect of rates of change in climate and LULC on the rate of change of species richness and assemblage dissimilarity in five ecoregions during a thirty-year period in the United States. Specifically, we hypothesized the following: 1) The rate of change in environmental variables (by which we mean climate, LULC, or both) will be associated with rates of change in all levels of biodiversity, and their components. As to which environmental driver is more strongly associated with changes in different biodiversity levels, based on the limited literature available, we hypothesize that 2) the relative importance of climate change and LULC change will be dependent on the ecoregion.

## Methods

### Data

#### Biodiversity rate of change data.

We used the rates of change in the species richness and assemblage dissimilarity of North American breeding birds across 30 years (1990–2019) obtained by [42]. We obtained data from the North American Breeding Bird Survey (BBS, [47]. We selected BBS routes in the conterminous United States sampled once a year in the period 1990–2019 that were sampled for more than 24 years, eliminating those that were not sampled in two or more consecutive years. Then, we retained only one BBS route per 10 × 10 km map grid cell by randomly eliminating one of two sampling units closer than 15 km (‘spThin’ R package, [48]. As in [42], this analysis included a total of 571 BBS routes: 309 on the Eastern Temperate Forest, 95 on the Great Plains, 61 on the Northern Forests, 56 on the Northwestern Forested Mountains, and 50 on the North American Deserts. Fig 1 in [42] illustrates the geographic distribution of the 571 BBS routes included in the study. We used the same exclusion and retention criteria as in [42] including in the analysis a total of 245 bird species (Table S1 in [42]). We converted the species abundances reported in the BBS to presence-absence data. We calculated the rates of change of species richness, total assemblage dissimilarity and its components as in [42].

#### Climatic data.

The climatic data were obtained from the Parameter-elevation Regressions on Independent Slopes Model (PRISM) datasets for the years 1990–2019 (PRISM Climate Group 2014; [49]. For this study, we selected climatic variables known to limit the distribution of birds physiologically [50–52]: mean precipitation (mm) minimum temperature(°C), and maximum temperature (°C). Each year, we calculated the mean values of these variables for the breeding season (i.e., May 1st to July 31st) using the methodology described in Supporting Information (S1 File).

#### LULC data.

To calculate the rate of LULC change between 1990 and 2019, we utilized the “Modeled Historical Land Use and Land Cover for the Conterminous US: 1938-1992” [53] and the scenario A1B of the “Conterminous United States Land Cover Projections - 1992 to 2100” [54] data sets. A detailed description of these data sets and the rationale for the selection of scenario A1B is provided in the Supporting Information (S1 File). We grouped the annual layers and retained for the analysis six combined layers containing the proportion of LULC per map cell for: barren land, crop/pasture, forest (deciduous forest + evergreen forest + mixed forest), grassland/shrubland, urban, and wetland (herbaceous wetland + woody wetland). S1 Fig in S1 File shows the methodology we used to obtain the grouped annual LULC layers.

#### Elevation data.

We included elevation in the analysis to account for its potential effect on biodiversity gradients. We used the North American Elevation map at 1 km resolution [55] to extract the elevation of each bird assemblage. Elevation information is given in meters, with values smaller than/ at/ above zero indicating below/ at/ above sea level, respectively.

To work with all data sets in the same spatial projection, we re-projected the environmental and biodiversity data to the Lambert Azimuthal Equal Area projection. We joined the annual climatic and land conversion layers and extracted the environmental information for each bird assemblage geographic coordinates.

### Rate of change in environmental conditions

**Fig 1:**
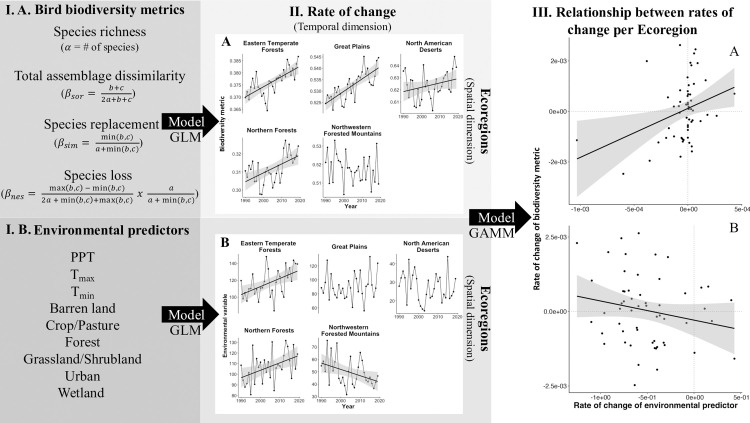
Methodological approach followed in the study. Step **I.**A: Four bird biodiversity metrics (response variables) were assessed across five ecoregions of the United States: one for species richness, and three for assemblage dissimilarity (total assemblage dissimilarity: Sorensen index, Species replacement: Simpson Index, and Species loss: Nestedness index). Step **I.**B: Predictor variables. We analyzed the relationship between the rate of change of each of the four biodiversity indices in time (Step **II.**A) to the rate of change of environmental variables (Step **II.**B) with generalized additive models (Step **III)**. In the equations in Step **I.**A: a = # species common to both sites, b = # species that occur in the first site but not in the second and c = # species that occur in the second site but not in the first (Baselga, 2010).

For each avian assemblage, we extracted the mean value of each annual climate and LULC map using three buffer sizes: 12.5 km, 25 km, and 50 km. We performed this step with the aim of accounting for potential effects that the scale of the analysis could have on the relationship between the rate of change in environmental variables and the rate of change of richness and assemblage dissimilarity [56]. At each buffer size, we calculated the rate of change of each environmental variable at each BBS route as the slope of the linear regression of the mean variable value with time (i.e., *mean value ~Year*) for the period 1990–2019 (Fig 1B II.). A positive slope indicated an increase in mean precipitation, minimum or maximum temperature, or proportion of LULC with time. A negative slope indicated a decrease in mean precipitation, minimum or maximum temperature, or proportion of LULC with time.

### Effect of the rate of environmental change on the biodiversity of species assemblages

We built a generalized additive mixed-effect regression model (GAMM) for each assemblage dissimilarity metric (i.e., total assemblage dissimilarity, species replacement, and species loss) and species richness in each ecoregion (gamm, “mgcv” R package, [57]. This type of model is frequently used in ecology to model non-linear and non-monotonic relationships [58]. GAMMs were fitted with a Gaussian distribution of errors and an identity link. We utilized GAMMs instead of Generalized Additive Models to account for spatial autocorrelation among the data points. The bird data were collected across space, which can result in biodiversity indices calculated from routes close together being more similar than those from routes further apart. To account for the possible spatial autocorrelation, we included a Gaussian spatial correlation structure of the form *~ Latitude + Longitude* in the models.

We used the rates of change of the assemblage dissimilarity metrics (log-transformed) and species richness as the response variables and the rate of change of climatic and LULC variables as predictors (all as smooth terms in the GAMMs). Dissimilarity metric values were log-transformed to standardize the variance. For each GAMM in each ecoregion, we reduced the number of predictor variables by excluding the LULC variable with the lowest mean proportion of LULC across the study period and the variables showing a correlation coefficient greater than 0.6. We included elevation as a control variable in the models as differences in biodiversity are associated with altitudinal gradients [59]. We built GAMMs for each buffer size (12.5 km, 25 km, and 50 km).

For all models, we used a significance level of α = 0.05 to determine the significance of predictor variables. Model diagnostics were performed through visual inspection of residual plots (“mgcv” R package, [57]). Most of the residuals’ Q-Q plots showed little or no deviations from the normality assumption. Exceptions were some GAMMs for the species replacement of Great Plains, and the species loss of Eastern Temperate Forests (depending on the resolution of the buffer used). In general, the residual versus predicted plots did not show any pattern. Exceptions were some GAMMs that showed heteroskedasticity in the residual versus predicted plots for the species richness, species replacement and/ or species loss (depending on the resolution of the buffer used). In these instances, there was some variability in the data that was not captured by the fitted GAMMs.

In the main text, we mainly present the scenario with the intermediate buffer resolution (25 km). Differences between models across buffer sizes are described in the Supporting Information and S1-S7 Tables in S1 File.

We assessed the relative importance of climate change and LULC change per ecoregion by comparing the number of climatic variables to the number of LULC variables that had a significant association with the biodiversity metrics analyzed in the GAMMs. Since the total number of climate predictor variables included in the GAMMs was lower than the total number of LULC predictor variables, we performed the comparison using proportions (e.g., 1 of 3 climatic variables or 3 of 5 LULC variables were significant predictors).

## Results

### Effect of the rate of change of the environment on biodiversity across ecoregions

On one hand biotic heterogenization and changes in richness of avian assemblages were linked to changes in climate and LULC. On the other hand, biotic homogenization and changes in the components of total assemblage dissimilarity were mostly linked to changes in LULC.

**Fig 2:**
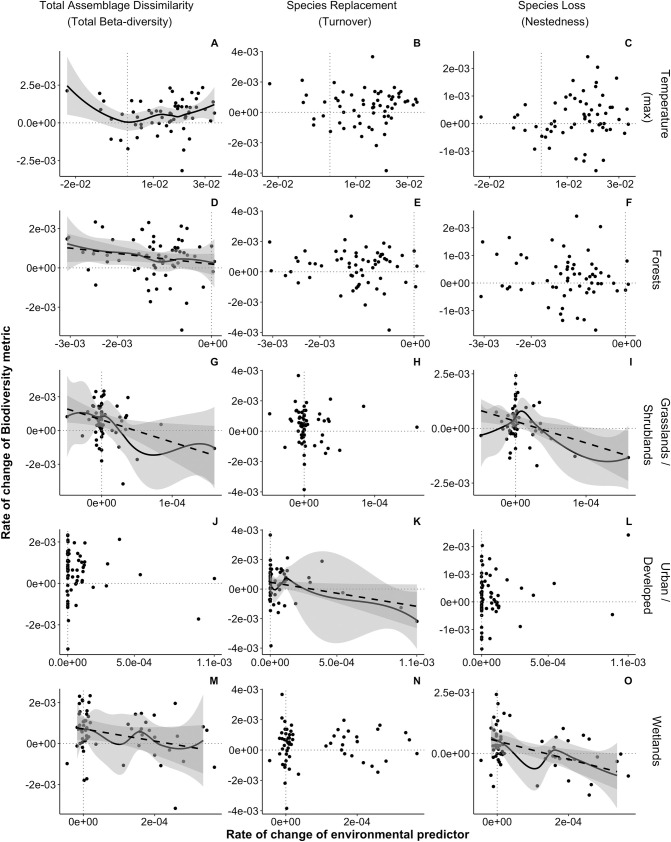
Relationship between the rate of change of assemblage dissimilarity with the rate of change of the environment in the US Northern Forests (1990–2019). Significant linear slopes are represented with dashed lines. Significant smooth terms are represented with solid lines.

**Fig 3:**
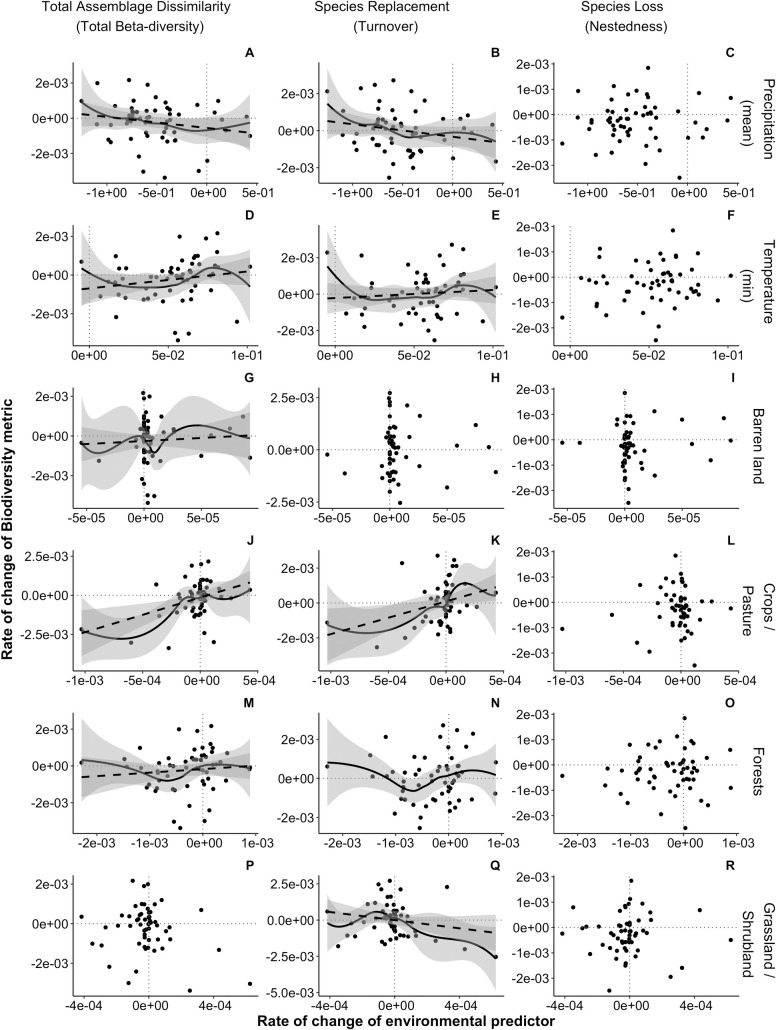
Relationship between the rate of change of assemblage dissimilarity with the rate of change of the environment in the US Northwestern Forested Mountains (1990–2019). Significant linear slopes are represented with dashed lines. Significant smooth terms are represented with solid lines.

Changes in biotic heterogenization were not driven by the same environmental factors from one ecoregion to another. Climatic changes were associated with changes in biotic heterogenization: rapid within-ecoregion heterogenization of avian assemblages was associated with both fast declines and increases in maximum temperature (Northern Forest; Table 1, Fig 2A), as well as with fast precipitation declines and rapid increase in minimum temperature (Northwestern Forested Mountains; Table 1, Fig 3A and 3B, 3D and 3E, respectively). Biotic heterogenization was also related to rapid changes in LULC. Northern Forests rapid assemblage heterogenization was related to fast forests declines (Table 1, Fig 2D). In the Northwestern Forested Mountains rapid assemblage heterogenization through species replacement was largely related to declines in forest faster than approximately −4.13x10^-4^ per unit time and increases in forest (though the effect size was small, Fig 3M), as well as rapid increases in barren land (Table 1, Fig 3M and 3N for change in forest, and G for change in barren land).

**Table 1:** GAMMs relating the rate of change of assemblage dissimilarity metrics with the rate of change of the environment in five ecoregions of the US at a resolution of 25 km (1990–2019).

Ecoregion	Biodiversity metric	PPT (mm)	T_max_ (^o^C)	T_min_ (^o^C)	Barren land	Crop/Pasture	Forest	Grassland/Shrubland	Urban	Wetland	Elevation (m)	R^2^
Northern Forest (*N* = 61)	Total dissimilarity	– (-)	– (**)	– (-)	NA	– (-)	−2.9* (*)	−3.2* (*)	– (-)	−4.7** (**)	– (-)	0.33
	Species replacement	– (-)	– (-)	– (-)	NA	– (-)	– (-)	– (-)	−3.4^ (*)	– (-)	– (-)	0.07
	Species loss	– (-)	– (-)	– (-)	NA	– (-)	– (-)	−2.9** (**)	– (-)	−4.8*** (***)	– (-)	0.30
Northwestern Forested Mountains (*N* = 56)	Total dissimilarity	−6.7*** (***)	– (-)	3.3* (*)	3.3* (*)	3.6* (*)	4* (*)	– (-)	NA	– (-)	NA	0.27
	Species replacement	−6.8** (**)	– (-)	−35** (***)	– (-)	5* (***)	– (***)	−3* (**)	NA	– (-)	NA	0
	Species loss	No term was significant										0
Great Plains (*N* = 95)	Total dissimilarity	– (-)	– (-)	– (-)	NA	NA	NA	– (-)	– (-)	– (-)	– (*)	0.07
	Species replacement	– (-)	– (-)	– (-)	NA	NA	NA	– (-)	– (-)	−3.5* (*)	– (-)	0.02
	Species loss	– (-)	– (-)	– (-)	NA	NA	NA	– (-)	– (-)	2.6* (*)	−2.2* (*)	0.12
North American Deserts (*N* = 50)	Total dissimilarity	No term was significant										0.06
	Species replacement	– (-)	NA	– (-)	– (-)	– (-)	– (*)	– (-)	NA	NA	5* (*)	0.10
	Species loss	– (-)	NA	– (-)	−3.6* (*)	– (-)	– (-)	– (-)	NA	NA	−3.1^^^ (*)	0.13
Eastern Temperate Forest (*N* = 309)	Total dissimilarity	– (-)	– (-)	– (-)	NA	– (-)	– (-)	– (-)	NA	– (-)	−2.7*** (***)	0.08
	Species replacement	−2.4** (**)	– (-)	– (-)	NA	– (-)	– (-)	– (-)	NA	– (-)	−1.9** (**)	0.04
	Species loss	2.1* (*)	– (-)	– (-)	NA	– (-)	– (-)	– (-)	NA	– (-)	– (-)	0.04

We show the linear model coefficients of variables with a significant effect and the significance of smooth terms between parenthesis for all variables. Linear model coefficients are on the scale of x10^-4^. We also present the proportion of the variability the model explains (Adjusted R^2^) and the number of BBS routes included in the analyses (*N*). Significant *P*-values are represented as follow: ***P < 0.001, **P < 0.01, *P < 0.05. Marginally significant *P*-values are represented as ^*P* < 0.065. Non-significant effects are denoted with a single dash (-) and predictor variables not included in the model are denoted with NA.

Bird assemblage homogenization was mainly associated with changes in LULC. In the Northern Forest, rapid homogenization through lowered species loss was related to fast increases in grassland/shrubland and wetland (Fig 2G, and 2I, and 2M and 2O). Although grassland/shrubland and wetland are not large proportions of the overall forest LULC type, the observed changes were quite rapid. In the Northwestern Forested Mountains, rapid assemblage homogenization was linked to rapid declines in crop/pasture (Fig 3J and 3K). In this ecoregion, rapid homogenization through slowed species replacement was also associated with fast increase in precipitation (Fig 3A and 3B). Rapid homogenization was found at high elevations in the Eastern Temperate Forest (driven by slowed-down species replacement rates; Table 1, S2D Fig and S2E Fig in S1 File) and in the Great Plains (driven by slowed-down species losses; Table 1, S3D Fig and S3F Fig in S1 File).

For the most part, changes in the components of total assemblage dissimilarity were related to changes in LULC. The single exception is that slowed species replacement coupled with fast species loss were associated with rapid increases in precipitation in the Eastern Temperate Forest (Table 1, S2B and S2C Fig in S1 File). Rapid species loss coupled with slower species replacement was also linked to rapid increases in wetland in the Great Plains (Table 1, S3B and S3C Fig in S1 File). Conversely, rapid species replacement rates coupled with slower species loss were found at high elevations in the North American Deserts (Table 1, S4H and S4I Fig in S1 File). Rapid species replacement was associated with fast decreases in forest in bird assemblages (*N*  = 13) located in areas with 30% or more of forest cover (averaged between 1990–2019) within the North American Deserts (S4E Fig, S8 Table in S1 File), while slower species replacement was related to fast increases in urban LULC in the Northern Forest (Fig 2K). Slowed species loss was linked to rapid increases in barren land in the North American Deserts (Table 1, S4C Fig in S1 File).

At the local level, rapid increases in species richness were associated with rapid increases in maximum temperature (Eastern Temperate Forests; Table 2, S5A Fig in S1 File) and intermediate rates of precipitation declines (Northwestern Forested Mountains; Table 2, S5D Fig in S1 File). Rapid species richness increases were also related to fast increases in urban LULC (Northern Forests; Table 2, S5C Fig in S1 File) and to fast declines in crop/pasture, fast increases in forest, and rapid changes in grassland/shrubland in any direction (Northwestern Forested Mountains; Table 2, S5D-S5F, S5H Fig in S1 File). Rapid species richness declines were linked to rapid declines in forests (Northern Forests and Northwestern Forested Mountains; Table 2, S5B, S5E Fig in S1 File) and to rapid warming of the minimum temperature (Northwestern Forested Mountains; Table 2, S5I Fig in S1 File). For Great Plains and North American Deserts, our models did not detect an association between rate of change in species richness and rate of change in environmental variables (Table 2).

**Table 2:** GAMMs relating the rate of change of species richness with the rate of change of the environment in five ecoregions of the US at a resolution of 25 km (1990–2019).

Ecoregion	PPT (mm)	T_max_ (^o^C)	T_min_ (^o^C)	Barren land	Crop/ Pasture	Forest	Grassland/ Shrubland	Urban	Wetland	Elevation (m)	R^2^
Northern Forest (*N* = 61)	– (-)	– (-)	– (-)	NA	– (-)	0.1* (*)	– (-)	0.1* (*)	– (-)	– (-)	0.17
Northwestern Forested Mountains (*N* = 56)	– (***)	– (-)	−0.09** (**)	– (-)	−0.07* (*)	– (*)	0.05^ (*)	NA	– (***)	NA	0.03
Great Plains (*N* = 95)	No term was significant										0
North American Deserts (*N* = 50)	No term was significant										0
Eastern Temperate Forest (*N* = 309)	– (-)	0.06** (**)	– (-)	NA	– (-)	– (-)	– (-)	NA	– (-)	– (-)	0.02

We show the linear model coefficients of variables with a significant effect and the significance of smooth terms between parenthesis for all variables. We also present the proportion of the variability the model explains (Adjusted R^2^) and the number of BBS routes included in the analyses (*N*). Significant *P*-values are represented as follow: ***P < 0.001, **P < 0.01, *P < 0.05. Marginally significant *P*-values are represented as ^*P* < 0.065. Non-significant effects are denoted with a single dash (-) and predictor variables not included in the model are denoted with NA.

#### Predictive power of dissimilarity models and species richness models across geographical scales.

We present values of the amount of variance in the biodiversity metrics explained by the environmental predictors (R^2^) for each model and resolution in Table 3. There was some variability in predictive power of the different models.

**Table 3:** Adjusted R^2^ across buffer resolutions for all GAMMs.

Ecoregion	Resolution (km)	Total assemblage dissimilarity	Species replacement	Species loss	Species Richness
Eastern Temperate Forest	12.5	0.09	0.03	0.05	0.01
	25	0.08	0.04	0.04	0.02
	50	0.09	0.03	0.03	0.03
Great Plains	12.5	0.08	0.02	0.12	0
	25	0.07	0.02	0.12	0
	50	0.12	0.06	0.14	0.06
North American Deserts	12.5	0	0.11	0	0
	25	0.06	0.10	0.13	0
	50	0.53	0.04	0.01	15
Northern Forests	12.5	0.27	0.06	0.19	0.17
	25	0.33	0.07	0.30	0.17
	50	0.30	0	0.32	0.14
Northwestern Forested Mountains	12.5	0.38	0.02	0	0.21
	25	0.27	0	0	0.03
	50	0.47	0.10	0	0

The predictive power of the models of species richness was consistently lower than that of the models for total assemblage dissimilarity (Table 3). Further, more environmental predictors were significantly associated with the rate of change of total assemblage dissimilarity than of species richness, with the exception of the model of species richness in the Northwestern Forested Mountains (Tables 1 and 2). In comparison to the aforementioned models, the predictive power of models for total assemblage dissimilarity in the Eastern Temperate Forest, Great Plains, and North American Deserts, and of models for species replacement and species loss, was lower (Table 3).

The results of the sensitivity analysis showed that the amount of variance in the four biodiversity metrics studied that was explained by the environmental predictors varied with the geographical scale of the analysis (Table 3). For instance, while the variance explained of total assemblage dissimilarity for the Northwestern Forested Mountains was 27% when the scale of the predictors was tabulated for 25 km sampling areas, the variance explained was 38% for 12.5 km and 47% for 50 km sampling areas.

### Relative importance of climate and LULC as drivers of rate of change of biodiversity

**Fig 4:**
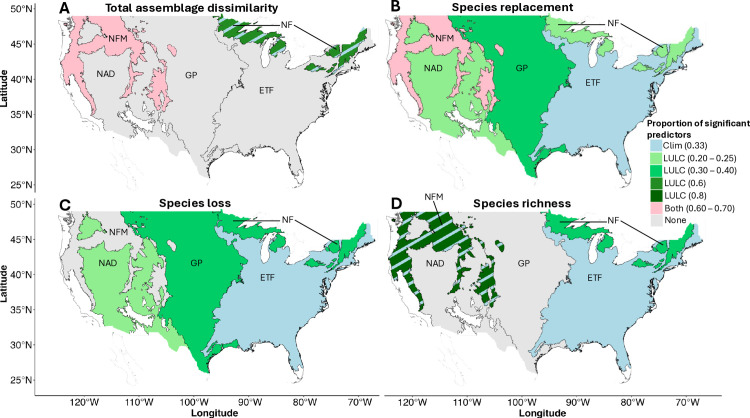
Relative importance of climate and LULC as drivers of rate of change of biodiversity in five ecoregions of the US (1990–2019). The legend shows the proportion of predictors per driver (i.e., climate, LULC, or both) for which the GAMMs showed a significant association with the biodiversity metrics. Hatched blue lines represent instances in which both climatic and LULC predictors were significant, but the proportion of significant LULC predictors surpassed the proportion of significant climatic predictors. NF: Northern Forest; NFM: Northwestern Forested Mountains, GP: Great Plains; NAD: North American Deserts; ETF: Eastern Temperate Forests.

The relative importance of climate change and LULC change varied by biodiversity metric and was ecoregion-dependent (Fig 4). As a general trend, LULC variables were more important predictors for all biodiversity metrics for the Northern Forest (Fig 4A-4D), for species replacement and loss in Great Plains and North American Deserts (Fig 4B and 4C), and for species richness in the Northwestern Forested Mountains (Fig 4D). Both climatic and LULC variables were equally important in predicting the total dissimilarity and species replacement in the Northwestern Forested mountains (Fig 4A and 4B). Climatic variables were the most important predictors for the species replacement, loss and richness of the Eastern Temperate Forests (Fig 4B-4D).

## Discussion

Our analyses spanned an extensive geographic region and a relatively long time period (30 years), revealing four main general results:

1. High elevation and high latitude ecoregions showed stronger relationships between biodiversity metrics and environmental factors where fast change in the environment was associated with fast biotic responses.
2. Species replacement and species loss showed association with environmental factors in opposite directions.
3. The rates of change in LULC were stronger predictors of rates of change in biodiversity than the rates of change in climate.
4. The rate of change in total assemblage dissimilarity was better predicted by the rate of change in the environment than was the rate of change of species richness.

### Effect of the rate of change of the environment on biodiversity across ecoregions

#### Increase of within-ecoregion assemblage dissimilarity (biotic heterogenization).

High latitude and high elevation places showed strongest relationships between biodiversity metrics and environmental drivers. Rapid within-ecoregion heterogenization of avian assemblages was associated with fast changes in climate and LULC in ecoregions at high latitudes or elevations such as the Northern Forest and the Northwestern Forested Mountains. These results point to the susceptibility of species assemblages at higher latitudes and elevations to environmental change also documented for other taxa [2,4,24,38,60,61]. Species at the edges of their thermal or habitat tolerances – due to species-specific physiological limits and breadth of ecological niches – will be particularly vulnerable to the changes we documented here.

For the two ecoregions with strongest relationships between the rates of change in biodiversity metrics and the environment, there were different environmental drivers and different outcomes (i.e., more species loss versus more species replacement). Below, we explore these two ecoregions further. The rate of within-ecoregion heterogenization of avian assemblages was impacted differentially by environmental variables in the Northern Forest and the Northwestern Forested Mountains. In general, the biological mechanism through which the biodiversity of bird assemblages was changing in response to environmental changes was also different between the two ecoregions, with species assemblages becoming subsets of richer assemblages in the Northern Forest and species being replaced in the Northwestern Forested Mountains.

Changes in temperature were associated with increased heterogenization in avian assemblages in these two ecoregions. Rapid changes in maximum temperature (Northern Forest) and minimum temperature (Northwestern Forested Mountains) were associated with rapid heterogenization of avian assemblages. Warming temperatures may have resulted in rapid increase in abundance and range areas of warm-dwelling species while cool-dwelling species decreased [62,63]. The heterogenizing effect of warming temperatures has been reported under current [24] and future [64] climate warming, in line with our results. These results likely indicate that some species were tracking climate change more rapidly or accurately than other species, as others have found [65,66]. The rate of biotic heterogenization may, however, not be high enough to keep the pace of the warming climate [67], since the stronger the climate warming, the more species’ responses may lag behind at range limits [68].

In the Northwestern Forested Mountains (but not the Northern Forest), increased water stress (i.e., declining precipitation) was associated with increased biotic heterogenization of avian assemblages. In the Northwestern Forested Mountains, rapid biotic heterogenization was also associated with rapidly drying locations. Increased water stress has been associated with range shifts to higher elevations, particularly when combined with changes in temperature [69]. Thus, species intolerant to low precipitation levels going rapidly locally extinct and species tolerant to low precipitation levels replacing them appear to be the mechanism behind the fast heterogenization of montane species assemblages undergoing decreases in precipitation. Decreases in precipitation have also been linked to population declines in European birds in mountainous regions due to phenological mismatches between resource availability and breeding season [70].

Rapid forest losses were generally associated with an accelerated rate of biotic heterogenization in the Northern Forests and the Northwestern Forested Mountains. However, the mechanisms behind this pattern were potentially different for each ecoregion. Rapid biotic heterogenization in the Northern Forests was likely driven by high rates of species loss (for example, loss of forest specialist species), while in Northwestern Forested Mountains, this process was possibly linked to high rates of species replacement (for example, replacement of forest specialists by species more tolerant to disturbed habitats). In the Northwestern Forested Mountains, the rate of change in total assemblage dissimilarity lagged behind the rate of change of forest loss, indicating that either extinction debt or buffering mechanisms may be operating here [4,71]. Although the consequences of forest loss on vertebrate biodiversity may be severe not only for already fragmented landscapes but also for relatively intact forested areas [72,73], the lagged response we found in the Northwestern Forested Mountains could be an indication of species assemblage resilience if critical habitat loss thresholds are not surpassed [14].

#### Decrease of within-ecoregion assemblage dissimilarity (biotic homogenization).

Here, we provide some context on how homogenization in this study might compare to homogenization in other studies. Because we are examining homogenization inside of ecoregions, it is not necessarily the case that the most generalized species will become the most common, as the ecoregion becomes more homogenized. It is a question of the scale of the analysis. For example, it could be that species typical of the ecoregion become more common within the ecoregion as homogenization progresses; these more common species might not be viewed as the most generalized, when comparing them to other species at a larger spatial scale. This is a nuance but is a result that is different from how other authors working at larger scales have conceptualized homogenization [4,61,74].

Rapid within-ecoregion homogenization of avian assemblages was mainly associated with fast changes in LULC. An exception was found in the Northwestern forested mountains, where fast increases in precipitation were associated with fast homogenization. Rapid increases in precipitation appear to be favoring ecoregion-specific species and slowing down their replacement by species tolerant of low precipitation levels, similar to results found by [62].

For Northern Forests and Northwestern Forested Mountains, the rapid change in LULC types less common than forest impacted the rate of change of total assemblage dissimilarity. For example, rapid increase of grassland/shrubland and wetland (within the Northern Forests) and rapid decreases in crop/pasture (within Northwestern Forested Mountains) were associated with accelerated rates of within-ecoregion biotic homogenization through the slowdown of species loss rates (Northern Forests) or declines in the species replacement rates (Northwestern Forested Mountains). Thus, rapid increases of natural LULC and declines of anthropogenic LULC appeared to be favoring habitat specialist species and slowing down their loss or replacement by disturbance-tolerant, widespread, generalist species. This finding highlights the importance of the heterogeneity of landscapes in maintaining biodiversity and providing resilience and stability of ecological processes against environmental change [75].

In the Eastern Temperate Forest and the Great Plains, the higher the elevation, the more similar the species assemblages within ecoregions. This occurred either because the species were less often replaced by others (Eastern Temperate Forests) or because they did not become a subset of richer species assemblages as fast as the species assemblages at lower elevation (Great Plains). These patterns may emerge from some species being restricted to certain areas and not colonizing whole gradients, likely due to environmental filtering linked to temperature and resource availability [76,77].

#### Effect of the rate of change of the environment on the components of assemblage dissimilarity.

The rate of change in the components of total dissimilarity was mainly related to changes in LULC, except for the Eastern Temperate Forest where precipitation was the most important predictor. In general, we found that the change in environmental predictors had opposite effects on the rates of change of species replacement and species loss in the Eastern Temperate Forest, Great Plains and North American Deserts. Opposite and/or independent responses of species replacement and species loss to predictor variables have been found for multiple taxa along elevational and latitudinal gradients [76,78,79], in agreement with what we found (S1-S3 Tables in S1 File). The pattern of decreased species replacement and increased species loss we identified in Eastern Temperate Forests and Great Plains is generally attributed to the presence of disturbance-tolerant or widespread species, and the local extinction of specialist or narrowly distributed species [80]. The pattern of increasing replacement rates and decreasing species loss rates with elevation found in North American Deserts is consistent with the upward range shifts of low-elevation species tracking their thermal niches [81].

Even small changes in the proportion of LULC can have large effects in relatively species-poor ecoregions. In North American Deserts, fast increases in barren land were associated with low rates of species loss, since likely the number of species present there was low to begin with due to the lack of vegetation characteristic of barren lands. Forest cover in this ecoregion is minimal, and its proportion is not changing significantly with time. Our models, however, detected that declines in forests in this ecoregion were associated with increased species turnover. Losing the habitat and resources that trees provide, the replacement of existing species by others better adapted to harsher environments is likely. In the Northern Forests, rapid increases in urban LULC (even if small in quantity) were related to lower rates of species replacement. In this case, we argue that, with the rapid habitat degradation that accompanies growing cities, there were fewer species to replace.

#### Effect of the rate of change of the environment on species richness.

We found significant relationships between species richness and the environmental variables for the Eastern Temperate Forest, the Northern Forests, and Northwestern Forested Mountains. In addition, the effects of climatic and LULC predictors on the rate of change of avian assemblage species richness, when significant, differed across ecoregions (similar to findings by [82]. We explore these relationships further, below.

Factors driving species richness were mainly changes in LULC. The only exception to this result was found in the Eastern Temperate Forests. There, fast increases in richness were associated with rapidly warming locations. This result is consistent with the northward range expansion of southerly-distributed, warm-affiliated species reported for eastern North America [62,83,84]. In contrast, other ecoregions saw increase in species richness driven by changes in LULC. In the Northern Forests, rapid increases in richness correlated with rapid increases in forests and urban LULC. An increase in richness with an increase in a particular LULC occurs when this change favors a particular group of species and immigration events exceed local extinction events [85]. Increases in forests may favor forest specialist birds with the increase in potential niches and habitat complexity. In contrast, rapid increases in urban LULC (even if small in quantity) may favor the increase of generalist, urban- or disturbance-tolerant species [86,87] in the avian assemblages of the Northern Forests. Decreases in crop/pasture were associated with fast increases in richness in the Northwestern Forested Mountains. The decrease in agricultural lands can potentially result in the increase of natural vegetation and the reduction of the effects of pesticide/fertilizer exposure, improving the quality of habitat for less disturbance-tolerant, late‐successional, and insectivore species [88–90].

#### Assemblage dissimilarity is better predicted by climate and/ or LULC than is species richness.

Our models performed reasonably well at predicting total assemblage dissimilarity for the northern and latitudinally more restricted ecoregions (Northern Forest and Northwestern Forested Mountains), though the better models were at the 25 km (Northern Forest) and 50 km (Northwestern Forested Mountains) scales. Models of the rate of change of species richness did not detect a significant relationship with the rate of change in the environment for the avian assemblages in the Great Plains and North American Deserts. In contrast, models of the rate of change of the three assemblage dissimilarity metrics detected significant relationship with at least one environmental predictor for all ecoregions. However, for some ecoregions, the models of the rates of change of the component of assemblage dissimilarity performed poorly since they explained a small percentage or none of the variance in the response variable. This may be an indication that the variation in the data was very small or that it would be explained by unmeasured variables (for example, [88], and the results for these models should be interpreted with caution. Interestingly, the variance in the response explained by the predictor variables was higher for the rate of change of total assemblage dissimilarity than for the rate of change of species richness. This is an indication that assemblage dissimilarity is more sensitive to changes in the environment than species richness, as other studies have also demonstrated [91,92]. Eventually, if the current rates of environmental change do not slow down, the effects of these changes will be reflected in the rate of change of species richness as well.

#### Interactions between the rates of change in climate and LULC.

Changes in climate and LULC can interact in their effects on biodiversity [25,26,93]. In this study, it was not possible to explore the effects of interactions among these two types of predictors due to data quantity limitations for some ecoregions. This restricts our findings to the additive effects of the rate of change in climate and LULC on the rate of change of biodiversity. Nevertheless, our results suggest that the effects of the rate of change in climate and LULC combined produce compositional changes in the bird assemblages in different directions. Rapid within-ecoregion heterogenization of species assemblages was driven by high rates of temperature change and rapid declines in precipitation, plus rapid declines in forests and grassland/shrubland. Homogenization within an ecoregion was faster in species assemblages experiencing rapid increases in precipitation, grassland/shrubland, and wetland or rapid declines in crop/pasture LULC. Consistent with our findings, previous studies have found that warming or drying climates combined with habitat loss or an increase in land use intensity severely affect species assemblages and population trends of insects and birds [25,26,93].

### Relative importance of climate and LULC as drivers of rate of change of biodiversity

In general, the rates of change in LULC were stronger predictors of bird assemblage rearrangement than rates of change in climate. In all ecoregions, there were significant relationships between at least one LULC variable and the rate of change of assemblage dissimilarity. The effects of the rate of change in climate, however, were only significant for the rate of change of species richness and assemblage dissimilarity in three of the five ecoregions. Other authors have found differential effects of climate and LULC on avian population trends as well [19,82].

In particular, the rate of change in LULC was a more important predictor of the rate of change of biodiversity in ecoregions dominated by low elevations (i.e., Great Plains and North American Deserts) and high latitudes (i.e., Northern Forest). Climate was more important for the rate of change of biodiversity in the Eastern Temperate Forest. Only in the mountainous region of the Northwestern Forested Mountains did the rate of change in both climate and LULC show similar importance in predicting biodiversity change. These results support the idea that the rate of change of climate and LULC affects species assemblages differentially by ecoregion [18,19]. A previous study in northern Europe reported that forest bird assemblages were more impacted by climate change [19], similar to our findings for the Eastern Temperate Forest. This same study found that farmland birds were impacted by changes in both climate and LULC [19], which contrasts with our findings for the Great Plains. This discrepancy in results may be linked to differences in species composition of the bird assemblages and the rate of change of the environment in different regions of the world. In any case, our results highlight the importance of considering multiple environmental factors and ecoregion-specific analysis in community ecology since threats to species assemblages can arise from changes in climate or LULC independently, or their combined effect [15], and vary per region [18,19].

## Conclusions

Changes in climate and/or LULC are driving the processes of community assembly and breakdown more than just driving the pattern of species richness and these assemblies and breakdowns can occur near one another within the same ecoregion. The more obvious changes are occurring in high altitude and high latitude ecoregions. For some ecoregions, components of total assemblage dissimilarity are responding to the environment in opposite ways. For all ecoregions, LULC was a stronger predictor of change in biodiversity metrics than was climate. Assemblage dissimilarity was better predicted (by the environment) than was species richness, despite the fact that species richness is the better studied phenomenon. It is only through examining trends in multiple biodiversity indices, meant to capture multiple levels of action (species richness versus assemblage dissimilarity) that we would be able to observe the complex and changing interrelationships between biodiversity (species richness and assemblage dissimilarity) and the environment (climate and LULC).

## Supporting information

S1 File Combined file containing the supporting information associated with this article. (DOCX)
