# Supplementary material for: The speed of change in climate and land cover is associated with the speed of biodiversity changes in avian assemblages of the United States
Source: PLoS One. 2025 Aug 29;20(8):e0330153. doi: 10.1371/journal.pone.0330153 (PMC12396716; doi:10.1371/journal.pone.0330153)
Supplement: S1 File — (DOCX) [file pone.0330153.s001.docx]

**Supporting Information**

METHODS

*Climatic data:*

The climatic data were obtained from the Parameter-elevation Regressions on Independent Slopes Model (PRISM) [1]). These datasets have a resolution of 4 km^2^ and cover the conterminous United States. Average monthly values of the climatic variables are calculated using all available station networks to arrive at the best estimate [1]. For this study, we used minimum temperature (°C), maximum temperature (°C) and precipitation (mm) for the breeding season for each year. We calculated the mean value per map cell of these climatic variables by aggregating each layer to a resolution of 10 x 10 km using the average value of the 4 km^2^ cells contained in the coarser resolution cell.

*Land use land cover data:*

For the land cover data, we utilized the “Modeled Historical Land Use and Land Cover for the Conterminous US: 1938-1992” [2] and the “Conterminous United States Land Cover Projections - 1992 to 2100” [3] data sets (with 250 m resolution). There were 14 LULC categories shared between these two data sets: Developed, three Forest types, Grasslands, Shrublands, Croplands, Hay / Pasture, two Wetland types, Open water, Barren, Ice / Snow, and Mining. We aggregated each layer to a resolution of 10x10 km, calculating the proportion of the corresponding LULC in the 100 km^2^ cell. Starting in 2006, the projected LULC data (Sohl et al. 2018) included four possible LULC scenarios corresponding to different developments of energy technologies (A1B, A2, B1, and B2). For this analysis, we selected the scenario A1B. We considered the A1B LULC scenario an appropriate selection since it represents intermediate increases in greenhouse gas emissions and land conversion in comparison to the other three scenarios available (see Tables 1 to 3 in [4] for definitions and comparison among scenarios in the year 2020).

RESULTS

Effect of scale on the relationship between the rate of change in environmental variables and the rate of change of assemblage dissimilarity.

*Assemblage dissimilarity (beta diversity and its components):* Increasing the size of the buffer at which the rates of change of environmental variables were calculated negatively affected the significance of the rate of change of mean precipitation as a predictor of the rate of change of species loss in Eastern Temperate Forest (Table S1) and species replacement rates in Northwestern Forested Mountains (Table S5). With increasing buffer size, the effect of the rate of change in crops also lost significance as a predictor of the rate of change of species replacement (Northwestern Forested Mountains – Table S5). In the Northern Forests, increasing the buffer size resulted in the loss of significance of the rate of change of urban LULC as a predictor of total assemblage dissimilarity and species replacement (turnover), and of the rate of change in wetlands as a predictor of total assemblage dissimilarity (Table S4). For Northwestern Forested Mountains, larger buffer sizes also negatively affected the significance of the rate of change in grasslands/shrublands as predictors of the rate of change of total assemblage dissimilarity and species replacement (Table S5).

On the other hand, increasing the size of the buffer at which the rates of change of environmental variables were calculated positively impacted the significance of the rate of change of mean precipitation as a predictor of total assemblage dissimilarity in North American Deserts (Table S3). In the case of the rate of change of maximum temperature, its effects as a predictor of the rate of change of species loss increased with larger buffer sizes in Northern Forests (Table S4). In the North American Deserts, the effect of the rate of change in crops significantly predicted the rate of change of total assemblage dissimilarity only at the larger buffer size (50 km). In the case of the rate of change of forest, the significance of its effect as a predictor of the rate of change of biodiversity metrics in the models generally increased with increasing buffer resolution (Tables S3 to S5). The significance of the rate of change of grasslands/shrublands was also positively affected with increasing buffer sizes when predicting the rate of change of total assemblage dissimilarity in the Northern Forests. Similarly for elevation, whose significance as a predictor of the rate of change of species loss (North American Deserts), and total assemblage dissimilarity (Northern Forests) increased with buffer size (Tables S3 and S4, respectively). For the rate of change in the proportion of barren land as a predictor of the rate of change of biodiversity metrics, the effect of buffer size was less consistent (Tables S3 and S5).

*Species richness (alpha diversity):* On the other hand, the significance of the rate of change in maximum temperature (Eastern Temperate Forest) and the proportion of forest (Northern Forest) on the rate of change in richness of the bird assemblages was not affected by increasing buffer size. Contrastingly, increasing the size of the buffer at which the rates of change of environmental variables were calculated negatively affected the significance of the rate of change of mean precipitation, minimum temperature and proportion of forests, wetlands, and crops/pastures as predictors of the rate of change of species richness in the Northwestern Forested Mountains (Table S6). Similar effect of increasing buffer size on the strength of the rate of change of urban LULC as a predictor of richness change was observed in the Northern Forests. For Great Plains and North American Deserts, increasing buffer size did not change the model results for any predictor variable (Table S7).

FIGURES

**
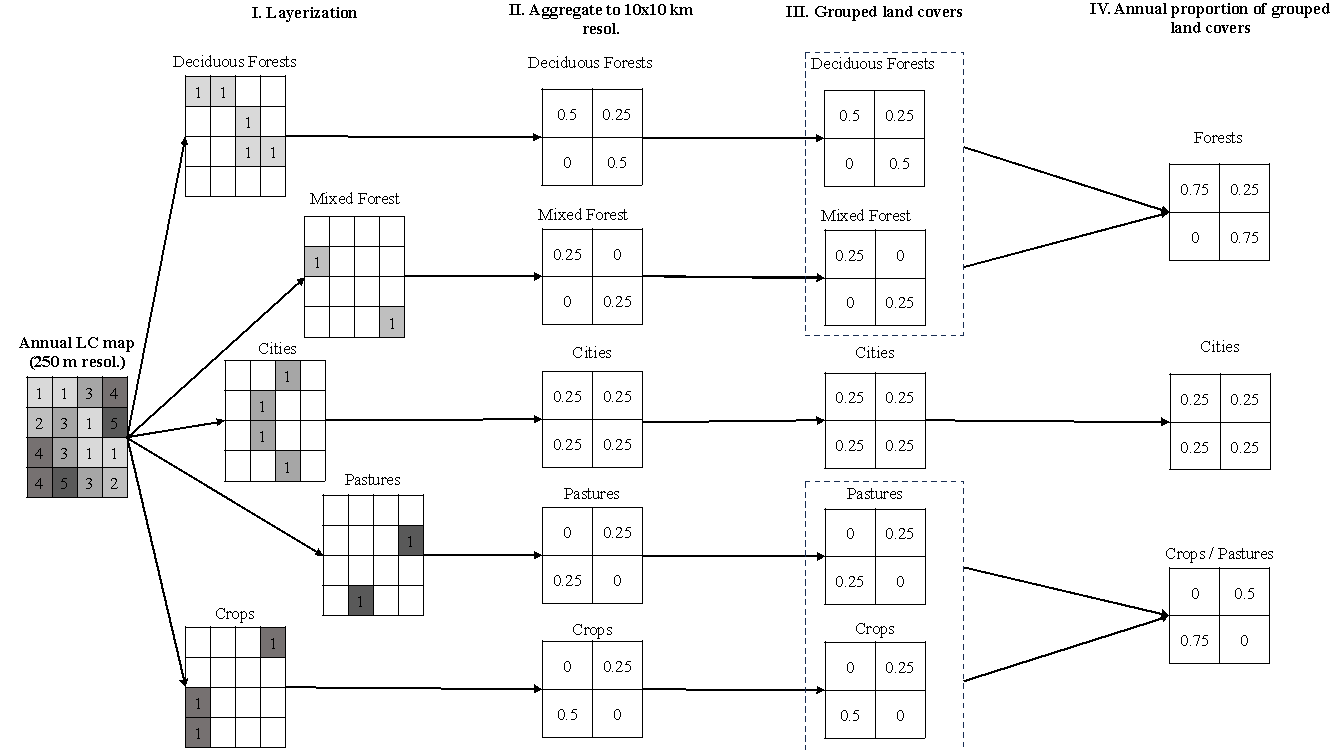
**

### **S1 Fig. The methodology followed to obtain the annual grouped LULC layers.** Starting with an annual LULC map at a 250 m resolution, the steps were: I) Layerization: Each LULC category was separated into its own layer. This process is carried out by assigning the value of one to cells if the LULC type in question was present in them and zero otherwise. II) Each layer was aggregated to a resolution of 10x10 km, calculating the proportion that the corresponding LULC was of the 100 km^2^ cell. This step yielded annual sets of individual layers, each layer indicating the proportion of the corresponding LULC type per cell. III) Grouped the 14 LULC layers into seven broader categories: Urban, Forests (Deciduous Forest, Evergreen Forest, Mixed Forest), Grassland-Shrubland, Cropland-Hay/Pasture, Wetlands-Water (Herbaceous Wetland, Woody Wetland), Barren, and Others (open water, Ice/Snow and Mining). IV) For each study year, the proportions of LULC type on these layers were added to obtain annual layers with the total proportion of the seven grouped LULC categories in each map cell.


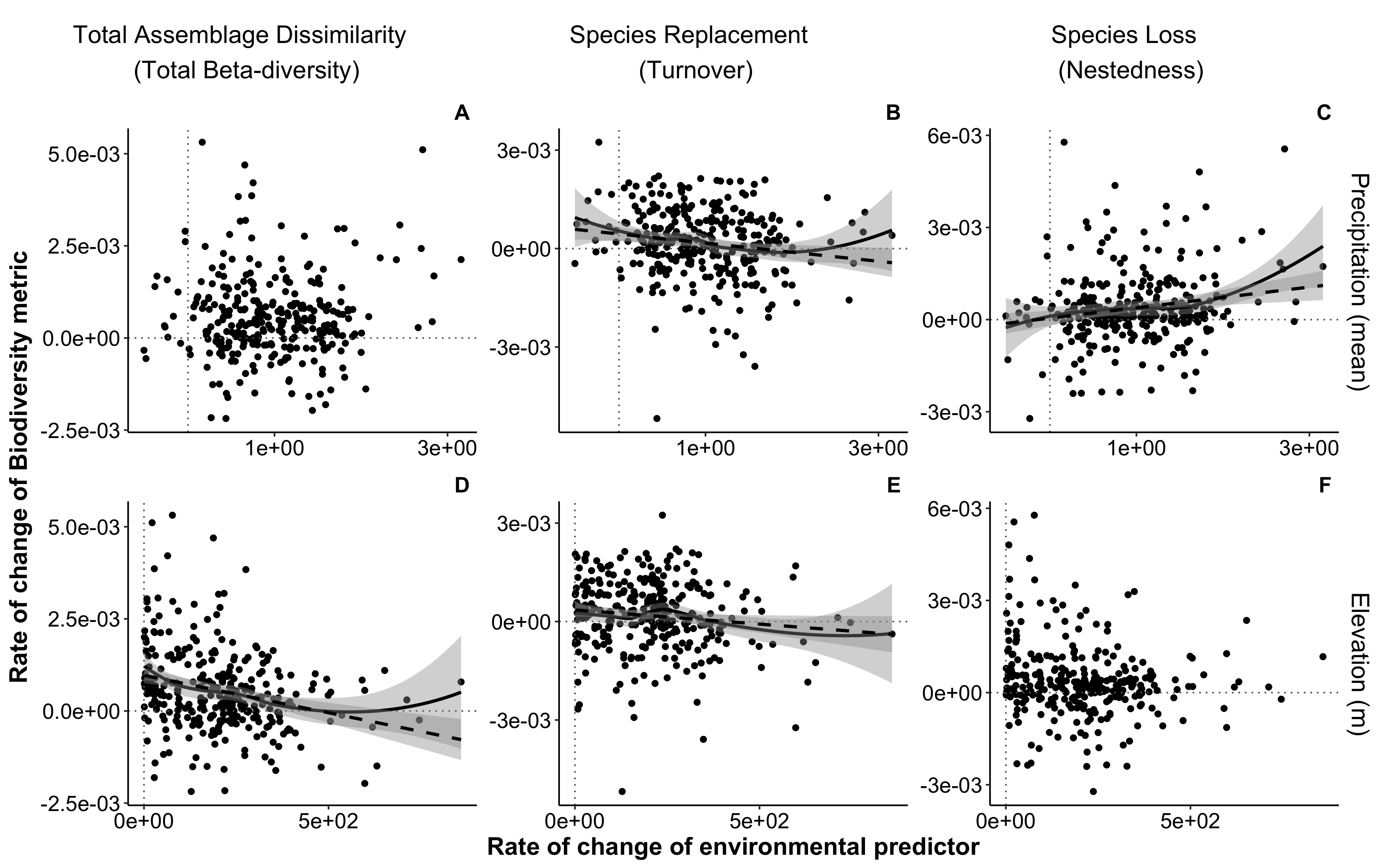


### **S2 Fig. Relationship between the rate of change of assemblage dissimilarity indices with the rate of change of the environment in the US Eastern Temperate Forests (1990 – 2019).** Significant linear slopes are represented with dashed lines. Significant smooth terms are represented with solid lines.


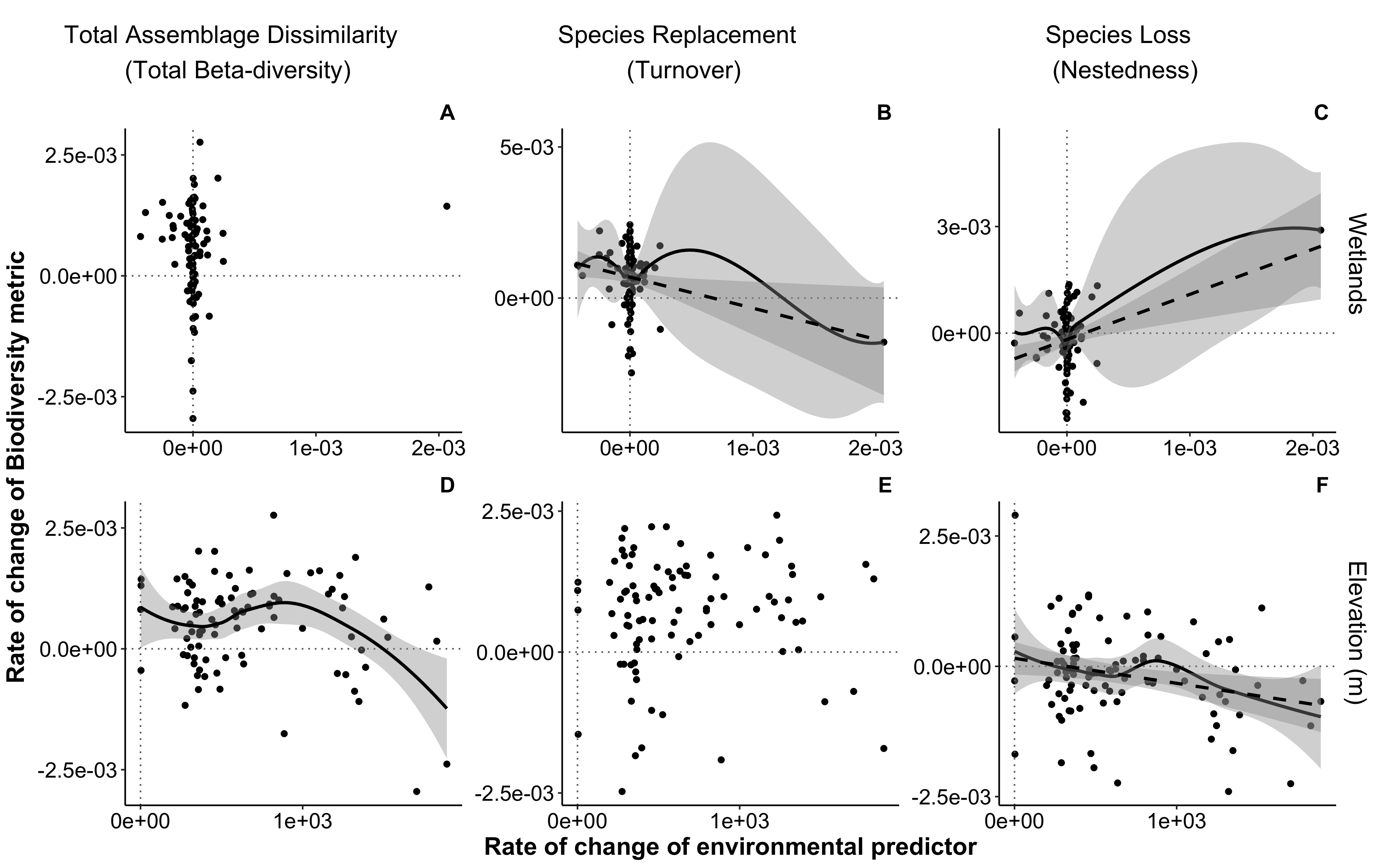


### **S3 Fig. Relationship between the rate of change of assemblage dissimilarity indices with the rate of change of the environment in the US Great Plains (1990 – 2019).** Significant linear slopes are represented with dashed lines. Significant smooth terms are represented with solid lines.


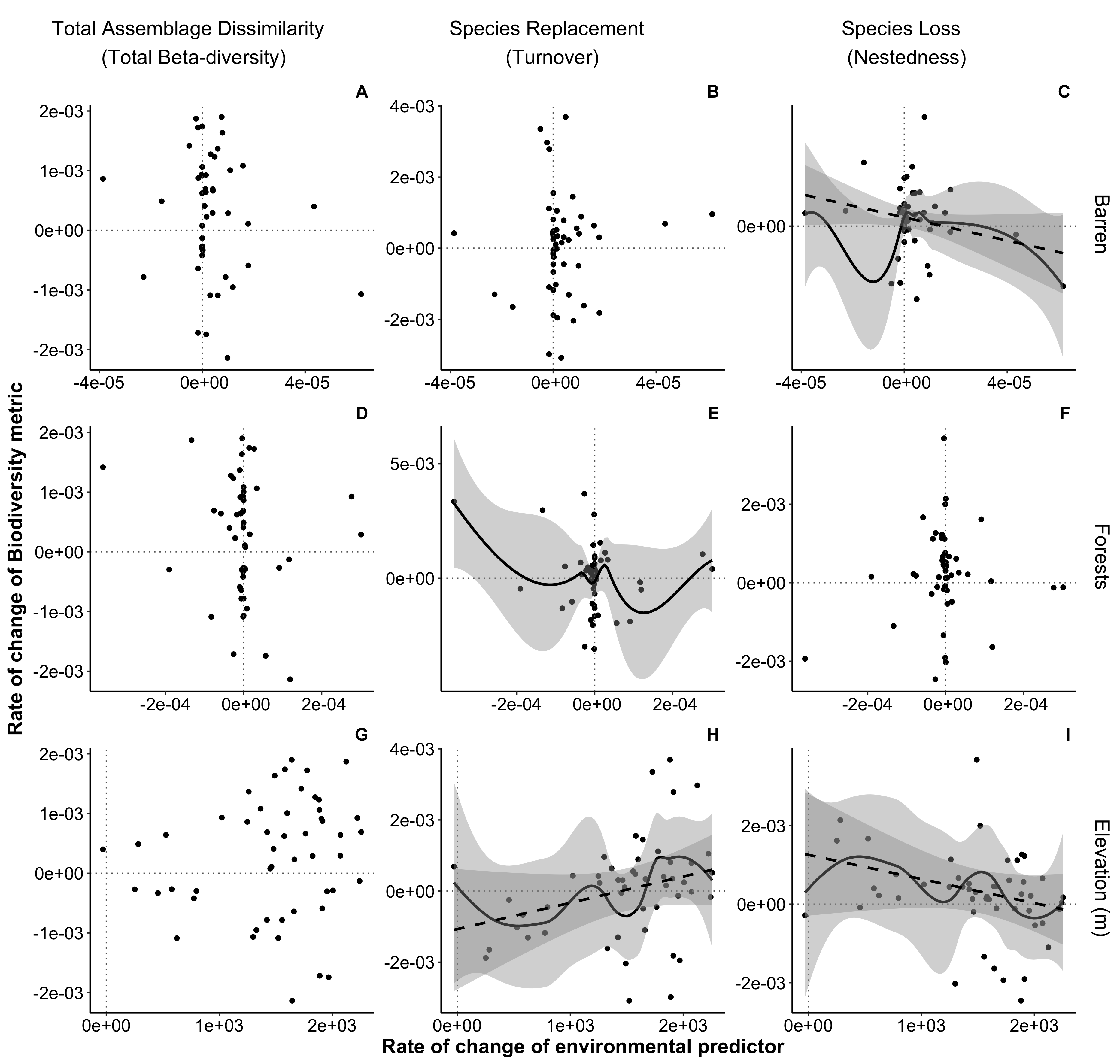


### **S4 Fig. Relationship between the rate of change of assemblage dissimilarity indices with the rate of change of the environment in the US North American Deserts (1990 – 2019).** Significant linear slopes are represented with dashed lines. Significant smooth terms are represented with solid lines.


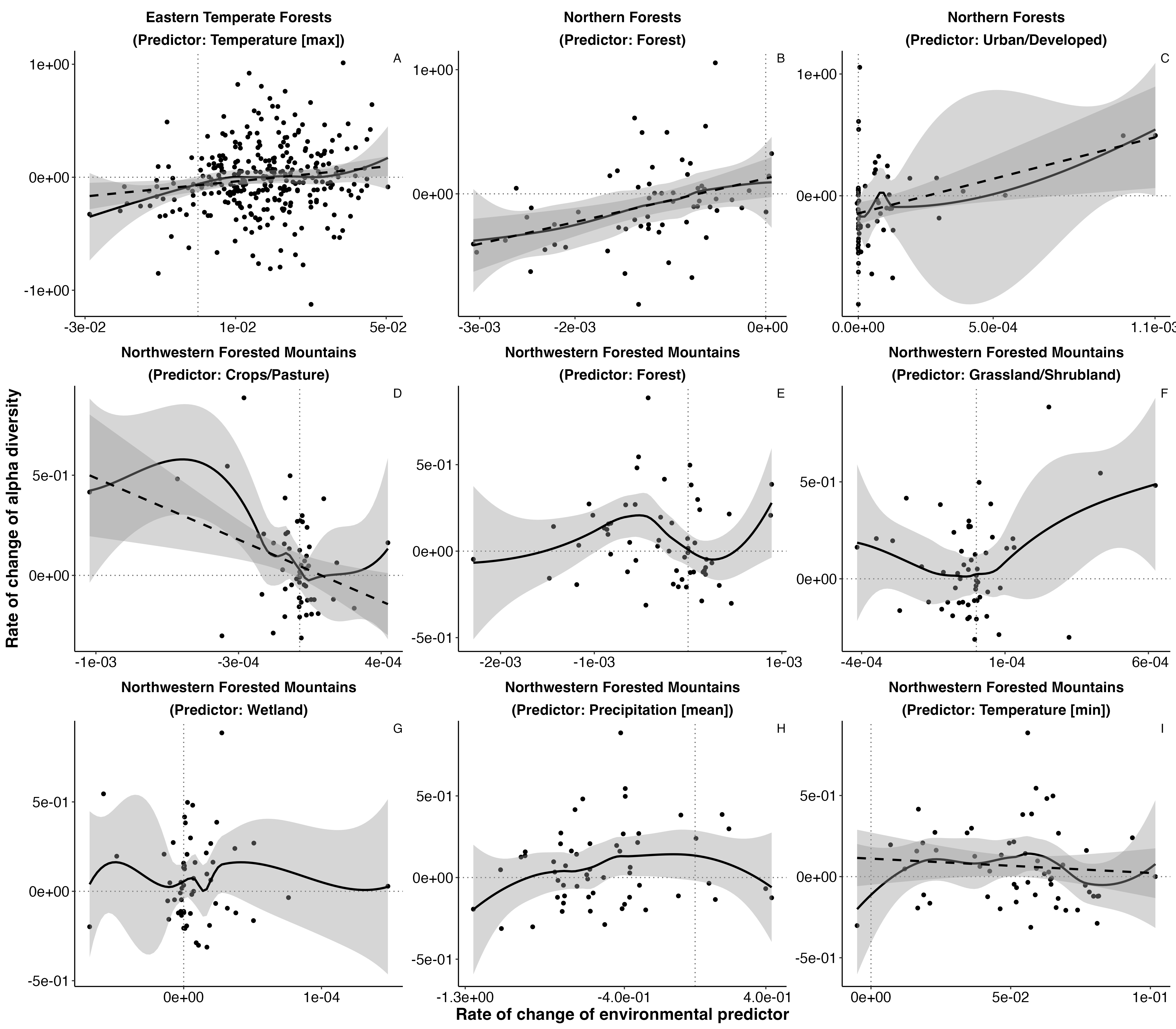


### **S5 Fig. Relationship between the rate of change of species richness (alpha diversity) with the rate of change of the environment in the US (1990 – 2019).** Significant linear slopes are represented with dashed lines. Significant smooth terms are represented with solid lines.

TABLES

**S1 Table. GAMMs relating the rate of change of assemblage dissimilarity with the rate of change of the environment in the US Eastern Temperate Forests (1990 – 2019).**

|  |  | **Total assemblage dissimilarity**  **(Total Beta diversity)** | | **Species replacement (Turnover)** | | **Species loss (Nestedness)** | |
| --- | --- | --- | --- | --- | --- | --- | --- |
| Predictor | Resolution (km) | Linear Slope | *P*-value  smooth terms | Linear Slope | *P*-value  smooth terms | Linear Slope | *P*-value  smooth terms |
| PPT _mean_ May-July | 12.5 | - | 0.39 | -2.1x10^-4^** | **0.004** | 2x10^-4^* | **0.01** |
|  | 25 | - | 0.17 | -2.4x10^-4^** | **0.002** | 2.1x10^-4^* | **0.01** |
|  | 50 | - | 0.11 | -2.6x10^-4^** | **0.002** | - | 0.08 |
| T_max_  May-July | 12.5 | - | 0.33 | - | 0.32 | - | 0.96 |
|  | 25 | - | 0.26 | - | 0.23 | - | 0.95 |
|  | 50 | -- | 0.15 | - | 0.36 | - | 0.55 |
| T_min_  May-July | 12.5 | -- | 0.70 | - | 0.75 | - | 0.49 |
|  | 25 | -- | 0.88 | - | 0.97 | - | 0.71 |
|  | 50 | - | 0.94 | - | 0.84 | - | 0.64 |
| Barren | 12.5 | -- | -- | -- | -- | -- | -- |
|  | 25 | -- | -- | -- | -- | -- | -- |
|  | 50 | -- | -- | -- | -- | -- | -- |
| Crops Pasture | 12.5 | -- | -- | -- | -- | -- | -- |
|  | 25 | - | 0.18 | - | 0.10 | - | 0.76 |
|  | 50 | - | 0.09 | - | 0.21 | - | 0.75 |
| Forests | 12.5 | - | 0.40 | - | 0.16 | - | 0.61 |
|  | 25 | - | 0.48 | - | 0.35 | - | 0.12 |
|  | 50 | - | 0.15 | - | 0.85 | - | 0.13 |
| Grassland Shrubland | 12.5 | - | 0.57 | - | 0.84 | - | 0.89 |
|  | 25 | - | 0.41 | - | 0.79 | - | 0.31 |
|  | 50 | - | 0.35 | - | 0.90 | - | 0.23 |
| Urban | 12.5 | - | 0.14 | - | 0.20 | - | 0.69 |
|  | 25 | -- | -- | -- | -- | -- | -- |
|  | 50 | -- | -- | -- | -- | -- | -- |
| Wetlands | 12.5 | - | 0.13 | - | 0.13 | - | 0.07 |
|  | 25 | - | 0.55 | - | 0.07 | - | 0.38 |
|  | 50 | - | 0.25 | - | 0.17 | - | 0.87 |
| Elevation (m) | 12.5 | -2.7x10^-4^*** | **0.0002** | -1.8x10^-4^** | **0.008** | - | 0.31 |
|  | 25 | -2.7x10^-4^*** | **0.0001** | -1.9x10^-4^** | **0.006** | - | 0.19 |
|  | 50 | -2.7x10^-4^*** | **<0.001** | -1.7x10^-4^* | **0.01** | - | 0.12 |
| R^2^ | 12.5 | 0.09  0.08  0.09 | | 0.03  0.04  0.03 | | 0.05  0.04  0.03 | |
|  | 25 |  |  |  |  |  |  |
|  | 50 |  |  |  |  |  |  |
| *N* | 309 | | | | | | |

We show the linear model coefficients of variables with a significant effect and the significance of smooth terms for all variables. We also present the proportion of the variability the model explains (Adjusted R^2^) and the number of BBS routes included in the analyses (*N*). Significant *P-*values for the smooth terms are highlighted in bold and significant *P-*values for the linear slope are represented as follows: ****P*<0.001, ***P*<0.01, **P*<0.05. Marginally significant *P-*values are represented as .*P*<0.065. Non-significant linear slopes are denoted with a single dash (-) and predictors not included in the model are denoted with a double dash (--).

**S2 Table. GAMMs relating the rate of change of assemblage dissimilarity with the rate of change of the environment in the US Great Plains (1990 – 2019).**

|  |  | **Total assemblage dissimilarity**  **(Total Beta diversity)** | | **Species replacement (Turnover)** | | **Species loss (Nestedness)** | |  |
| --- | --- | --- | --- | --- | --- | --- | --- | --- |
| Predictor | Resolution (km) | Linear Slope | *P-*value  smooth terms | Linear Slope | *P-*value  smooth terms | Linear Slope | *P-*value  smooth terms | |
| PPT _mean_ May-July | 12.5 | - | 0.81 | - | 0.96 | - | 0.70 | |
|  | 25 | - | 0.96 | - | 0.96 | - | 0.57 | |
|  | 50 | - | 0.68 | - | 0.17 | - | 0.96 | |
| T_max_  May-July | 12.5 | - | 0.44 | - | 0.18 | - | 0.33 | |
|  | 25 | - | 0.51 | - | 0.19 | - | 0.30 | |
|  | 50 | - | 0.28 | - | 0.17 | - | 0.32 | |
| T_min_  May-July | 12.5 | - | 0.79 | - | 0.24 | - | 0.83 | |
|  | 25 | - | 0.59 | - | 0.16 | - | 0.85 | |
|  | 50 | - | 0.95 | - | 0.10 | - | 0.39 | |
| Barren | 12.5 | -- | -- | -- | -- | -- | -- | |
|  | 25 | -- | -- | -- | -- | -- | -- | |
|  | 50 | -- | -- | -- | -- | -- | -- | |
| Crops Pasture | 12.5 | -- | -- | -- | -- | -- | -- | |
|  | 25 | -- | -- | -- | -- | -- | -- | |
|  | 50 | -- | -- | -- | -- | -- | -- | |
| Forests | 12.5 | -- | -- | -- | -- | -- | -- | |
|  | 25 | - | 0.21 | - | 0.23 | - | 0.73 | |
|  | 50 | - | 0.45 | - | 0.10 | - | 0.77 | |
| Grassland Shrubland | 12.5 | - | 0.45 | - | 0.64 | - | 0.73 | |
|  | 25 | - | 0.59 | - | 0.94 | - | 0.40 | |
|  | 50 | - | 0.91 | - | 0.74 | - | 0.40 | |
| Urban | 12.5 | - | 0.74 | - | 0.93 | - | 0.83 | |
|  | 25 | - | 0.89 | - | 0.43 | - | 0.26 | |
|  | 50 | - | 0.72 | - | 0.35 | - | 0.09 | |
| Wetlands | 12.5 | - | 0.53 | -2.5x10^-4^* | **0.02** | 3x10^-4^*** | **0.001** | |
|  | 25 | - | 0.67 | -3.5x10^-4^* | **0.01** | 2.6x10^-4^* | **0.02** | |
|  | 50 | - | 0.15 | -3.1x10^-4^* | **0.01** | - | **0.01** | |
| Elevation (m) | 12.5 | - | **0.01** | - | 1.00 | -1.8x10^-4^* | **0.03** | |
|  | 25 | - | **0.01** | - | 0.84 | -2.2x10^-4^* | **0.01** | |
|  | 50 | - | **0.01** | - | 0.57 | -2.2x10^-4^* | **0.02** | |
| R^2^ | 12.5 | \| 0.08 \| \| --- \| \| 0.07 \| \| 0.12 \| | | \| 0.02 \| \| --- \| \| 0.02 \| \| 0.06 \| | | \| 0.12 \| \| --- \| \| 0.12 \| \| 0.14 \| | |  |
|  | 25 |  |  |  |  |  |  |  |
|  | 50 |  |  |  |  |  |  |  |
| *N* | 95 | | | | | | |  |

### We show the linear model coefficients of variables with a significant effect and the significance of smooth terms for all variables. We also present the proportion of the variability the model explains (Adjusted R^2^) and the number of BBS routes included in the analyses (*N*). Significant *P-*values for the smooth terms are highlighted in bold and significant *P-*values for the linear slope are represented as follows: ****P*<0.001, ***P*<0.01, **P*<0.05. Marginally significant *P-*values are represented as .*P*<0.065. Non-significant linear slopes are denoted with a single dash (-) and predictors not included in the model are denoted with a double dash (--).

### **S3 Table. GAMMs relating the rate of change of assemblage dissimilarity with the rate of change of the environment in the US North American Deserts (1990 – 2019).**

|  |  | **Total assemblage dissimilarity**  **(Total Beta diversity)** | | **Species replacement (Turnover)** | | **Species loss (Nestedness)** | |
| --- | --- | --- | --- | --- | --- | --- | --- |
| Predictor | Resolution (km) | Linear Slope | *P*-value  smooth terms | Linear Slope | *P*-value  smooth terms | Linear Slope | *P*-value  smooth terms |
| PPT _mean_ May-July | 12.5 | - | 0.30 | - | 0.46 | - | 0.64 |
|  | 25 | - | 0.16 | - | 0.35 | - | 0.78 |
|  | 50 | - | **0.001** | - | 0.25 | - | 0.78 |
| T_max_  May-July | 12.5 | - | 0.96 | - | 0.47 | - | 0.41 |
|  | 25 | -- | -- | -- | -- | -- | -- |
|  | 50 | -- | -- | -- | -- | -- | -- |
| T_min_  May-July | 12.5 | - | 0.38 | - | 0.65 | - | 0.45 |
|  | 25 | - | 0.10 | - | 0.59 | - | 0.27 |
|  | 50 | - | 0.34 | - | 0.91 | - | 0.62 |
| Barren | 12.5 | - | 0.84 | - | 0.68 | - | 0.51 |
|  | 25 | - | 0.17 | - | 0.34 | -3.6x10^-4^* | **0.01** |
|  | 50 | - | **0.02** | - | 0.37 | - | 0.21 |
| Crops Pasture | 12.5 | - | 0.69 | - | 0.64 | - | 0.74 |
|  | 25 | - | 0.07 | - | 0.33 | - | 0.63 |
|  | 50 | 4.5x10^-4^*** | **0.0001** | - | 0.33 | - | 0.54 |
| Forests | 12.5 | - | 0.30 | - | 0.06 | - | 0.24 |
|  | 25 | - | 0.05 | - | **0.04** | - | 0.16 |
|  | 50 | - | **0.0002** | - | 0.11 | - | 0.65 |
| Grassland Shrubland | 12.5 | - | 0.36 | - | 0.20 | - | 0.51 |
|  | 25 | - | 0.41 | - | 0.21 | - | 0.36 |
|  | 50 | - | 0.87 | - | 0.43 | - | 0.62 |
| Urban | 12.5 | -- | -- | -- | -- | -- | -- |
|  | 25 | -- | -- | -- | -- | -- | -- |
|  | 50 | -- | -- | -- | -- | -- | -- |
| Wetlands | 12.5 | -- | -- | -- | -- | -- | -- |
|  | 25 | -- | -- | -- | -- | -- | -- |
|  | 50 | -- | -- | -- | -- | -- | -- |
| Elevation (m) | 12.5 | - | 0.30 | 4.4x10^-4^* | **0.03** | - | 0.05 |
|  | 25 | - | 0.11 | 5x10^-4^* | **0.01** | -3.1x10^-4^. | **0.04** |
|  | 50 | - | 0.16 | 6.2x10^-4^** | **0.00** | -3.8x10^-4^* | **0.03** |
| R^2^ | 12.5 | \| 0 \| \| --- \| \| 0.06 \| \| 0.53 \| | | \| 0.11 \| \| --- \| \| 0.10 \| \| 0.04 \| | | \| 0 \| \| --- \| \| 0.13 \| \| 0.01 \| | |
|  | 25 |  |  |  |  |  |  |
|  | 50 |  |  |  |  |  |  |
| *N* | 50 | | | | | | |

We show the linear model coefficients of variables with a significant effect and the significance of smooth terms for all variables. We also present the proportion of the variability the model explains (Adjusted R^2^) and the number of BBS routes included in the analyses (*N*). Significant *P-*values for the smooth terms are highlighted in bold and significant *P-*values for the linear slope are represented as follows: ****P*<0.001, ***P*<0.01, **P*<0.05. Marginally significant *P-*values are represented as .*P*<0.065. Non-significant linear slopes are denoted with a single dash (-) and predictors not included in the model are denoted with a double dash (--).

### **S4 Table. GAMMs relating the rate of change of assemblage dissimilarity with the rate of change of the environment in the US Northern Forests (1990 – 2019).**

|  |  | **Total assemblage dissimilarity**  **(Total Beta diversity)** | | **Species replacement (Turnover)** | | **Species loss (Nestedness)** | |
| --- | --- | --- | --- | --- | --- | --- | --- |
| Predictor | Resolution (km) | Linear Slope | *P*-value  smooth terms | Linear Slope | *P*-value  smooth terms | Linear Slope | *P*-value  smooth terms |
| PPT _mean_ May-July | 12.5 | - | 0.18 | - | 0.13 | - | 0.44 |
|  | 25 | - | 0.44 | - | 0.16 | - | 0.16 |
|  | 50 | -- | -- | -- | -- | -- | -- |
| T_max_  May-July | 12.5 | - | **0.02** | - | 0.15 | - | 0.39 |
|  | 25 | - | **0.003** | - | 0.09 | - | 0.13 |
|  | 50 | 2.9x10^-4^* | **0.03** | - | 0.77 | 2.2x10^-4^* | **0.02** |
| T_min_  May-July | 12.5 | -3.6x10^-4^* | **0.02** | - | 0.15 | - | 0.51 |
|  | 25 | - | 0.06 | - | 0.27 | - | 0.50 |
|  | 50 | -- | -- | -- | -- | -- | -- |
| Barren | 12.5 | -- | -- | -- | -- | -- | -- |
|  | 25 | -- | -- | -- | -- | -- | -- |
|  | 50 | -- | -- | -- | -- | -- | -- |
| Crops Pasture | 12.5 | - | 0.07 | - | 0.28 | - | 0.63 |
|  | 25 | - | 0.22 | - | 0.81 | - | 0.32 |
|  | 50 | - | 0.93 | - | 0.98 | - | 0.89 |
| Forests | 12.5 | - | 0.18 | - | 0.54 | - | 0.53 |
|  | 25 | -2.9x10^-4^* | **0.02** | - | 0.46 | - | 0.15 |
|  | 50 | -3.1x10^-4^* | **0.01** | - | 0.26 | - | 0.08 |
| Grassland Shrubland | 12.5 | - | 0.09 | - | 0.72 | -2.7x10^-4^* | **0.01** |
|  | 25 | -3.2x10^-4^* | **0.02** | - | 0.88 | -2.9x10^-4^** | **0.003** |
|  | 50 | -4.2x10^-4^** | **0.002** | - | 0.46 | -2.1x10^-4^* | **0.03** |
| Urban | 12.5 | -2.8x10^-4^* | **0.02** | -3.5x10^-4^* | **0.02** | - | 0.51 |
|  | 25 | - | 0.06 | -3.4x10^-4^. | **0.04** | - | 0.22 |
|  | 50 | - | 0.16 | - | 0.84 | - | 0.22 |
| Wetlands | 12.5 | -4.2x10^-4^* | **0.01** | - | 1.00 | -4.2x10^-4^** | **0.001** |
|  | 25 | -4.7x10^-4^** | **0.002** | - | 0.97 | -4.8x10^-4^*** | **<0.001** |
|  | 50 | - | 0.11 | - | 0.68 | -3x10^-4^** | **0.001** |
| Elevation (m) | 12.5 | - | 0.57 | - | 0.31 | - | 0.38 |
|  | 25 | - | 0.18 | - | 0.23 | - | 0.70 |
|  | 50 | - | **0.03** | - | 0.27 | - | 0.56 |
| R^2^ | 12.5 | \| 0.27 \| \| --- \| \| 0.33 \| \| 0.30 \| | | \| 0.06 \| \| --- \| \| 0.07 \| \| 0 \| | | \| 0.19 \| \| --- \| \| 0.30 \| \| 0.32 \| | |
|  | 25 |  |  |  |  |  |  |
|  | 50 |  |  |  |  |  |  |
| *N* | 61 | | | | | | |

We show the linear model coefficients of variables with a significant effect and the significance of smooth terms for all variables. We also present the proportion of the variability the model explains (Adjusted R^2^) and the number of BBS routes included in the analyses (*N*). Significant *P-*values for the smooth terms are highlighted in bold and significant *P-*values for the linear slope are represented as follows: ****P*<0.001, ***P*<0.01, **P*<0.05. Marginally significant *P-*values are represented as .*P*<0.065. Non-significant linear slopes are denoted with a single dash (-) and predictors not included in the model are denoted with a double dash (--).

### **S5 Table. GAMMs relating the rate of change of assemblage dissimilarity with the rate of change of the environment in the US Northwestern Forested Mountains (1990 – 2019).**

|  |  | **Total assemblage dissimilarity**  **(Total Beta diversity)** | | **Species replacement (Turnover)** | | **Species loss (Nestedness)** | |
| --- | --- | --- | --- | --- | --- | --- | --- |
| Predictor | Resolution (km) | Linear Slope | *P*-value  smooth terms | Linear Slope | *P*-value  smooth terms | Linear Slope | *P*-value  smooth terms |
| PPT _mean_ May-July | 12.5 | -5x10^-4^** | **0.003** | -4.4x10^-4^* | **0.02** | - | 0.98 |
|  | 25 | -6.7x10^-4^*** | **<0.001** | -6.8x10^-4^** | **0.002** | - | 0.58 |
|  | 50 | - | 0.07 | - | 0.18 | - | 0.56 |
| T_max_  May-July | 12.5 | - | 0.05 | - | 0.45 | - | 0.93 |
|  | 25 | - | 0.23 | - | 0.39 | - | 0.78 |
|  | 50 | -- | -- | -- | -- | -- | -- |
| T_min_  May-July | 12.5 | -1.5x10^-3^* | **0.002** | - | 0.98 | - | 0.38 |
|  | 25 | 3.3x10^-4^* | **0.03** | -3.5x10^-3^** | **<0.001** | - | 0.32 |
|  | 50 | - | 0.07 | - | 0.11 | - | 0.23 |
| Barren | 12.5 | - | 0.27 | - | 0.63 | - | 0.63 |
|  | 25 | 3.3x10^-4^* | **0.03** | - | 0.41 | - | 0.34 |
|  | 50 | 3.1x10^-4^* | **0.03** | - | 0.52 | - | 0.41 |
| Crops Pasture | 12.5 | -- | -- | -- | -- | -- | -- |
|  | 25 | 3.6x10^-4^* | **0.03** | 5x10^-4^* | **<0.001** | - | 0.65 |
|  | 50 | 3.7x10^-4^* | **0.01** | - | 0.21 | - | 0.52 |
| Forests | 12.5 | - | 0.53 | - | 0.32 | - | 0.90 |
|  | 25 | 3.9x10^-4^* | **0.02** | - | **<0.001** | - | 0.57 |
|  | 50 | 3.2x10^-4^* | **0.03** | - | 0.37 | - | 0.42 |
| Grassland Shrubland | 12.5 | - | **0.001** | - | **<0.001** | - | 0.24 |
|  | 25 | - | 0.47 | -3x10^-4^* | **0.01** | - | 0.98 |
|  | 50 | - | 0.57 | -- | 0.31 | - | 0.40 |
| Urban | 12.5 | -- | -- | -- | -- | -- | -- |
|  | 25 | -- | -- | -- | -- | -- | -- |
|  | 50 | -- | -- | -- | -- | -- | -- |
| Wetlands | 12.5 | - | 0.61 | - | 0.89 | - | 0.79 |
|  | 25 | - | 0.53 | - | 0.66 | - | 0.95 |
|  | 50 | -2.8x10^-3^* | **0.001** | - | 0.79 | - | 0.61 |
| Elevation (m) | 12.5 | -- | -- | -- | -- | -- | -- |
|  | 25 | -- | -- | -- | -- | -- | -- |
|  | 50 | -- | -- | -- | -- | -- | -- |
| R^2^ | 12.5 | \| 0.38 \| \| --- \| \| 0.27 \| \| 0.47 \| | | \| 0.02 \| \| --- \| \| 0 \| \| 0.10 \| | | \| 0 \| \| --- \| \| 0 \| \| 0 \| | |
|  | 25 |  |  |  |  |  |  |
|  | 50 |  |  |  |  |  |  |
| *N* | 56 | | | | | | |

We show the linear model coefficients of variables with a significant effect and the significance of smooth terms for all variables. We also present the proportion of the variability the model explains (Adjusted R^2^) and the number of BBS routes included in the analyses (*N*). Significant *P-*values for the smooth terms are highlighted in bold and significant *P-*values for the linear slope are represented as follows: ****P*<0.001, ***P*<0.01, **P*<0.05. Marginally significant *P-*values are represented as .*P*<0.065. Non-significant linear slopes are denoted with a single dash (-) and predictors variables not included in the model are denoted with a double dash (--)

### **S6 Table. GAMMs relating the rate of change of species richness with the rate of change of the environment in US Eastern Temperate Forests, Northern Forests, and Northwestern Forested Mountains (1990 – 2019).**

|  |  | Eastern Temperate Forests | | | Northern Forests | | | Northwestern Forested Mountains | | |
| --- | --- | --- | --- | --- | --- | --- | --- | --- | --- | --- |
| Predictor | Resolution (km) | Linear Slope | | *P*-value  smooth terms | Linear Slope | | *P*-value  smooth terms | Linear Slope | | *P*-value  smooth terms |
| PPT _mean_ May-July | 12.5 | - | | 0.80 | - | | 0.50 | - | | **<0.001** |
|  | 25 | - | | 0.58 | - | | 0.75 | - | | **<0.001** |
|  | 50 | - | | 0.23 | -- | | -- | - | | 0.09 |
| T_max_  May-July | 12.5 | 0.05* | | **0.02** | - | | 0.84 | - | | 0.50 |
|  | 25 | 0.06** | | **0.01** | - | | 0.53 | - | | 0.94 |
|  | 50 | 0.07** | | **0.001** | - | | 0.58 | -- | | -- |
| T_min_  May-July | 12.5 | - | | 0.16 | - | | 0.16 | -0.07** | | **0.01** |
|  | 25 | - | | 0.17 | - | | 0.19 | -0.09** | | **0.004** |
|  | 50 | - | | 0.18 | -- | | -- | - | | 0.65 |
| Barren | 12.5 | -- | | -- | -- | | -- | - | | 0.14 |
|  | 25 | -- | | -- | -- | | -- | - | | 0.35 |
|  | 50 | -- | | -- | -- | | -- | - | | 0.68 |
| Crops Pasture | 12.5 | -- | | -- | - | | 0.25 | -- | | -- |
|  | 25 | - | | 0.12 | - | | 0.63 | -0.07* | | **0.01** |
|  | 50 | - | | 0.08 | - | | 0.73 | - | | 0.17 |
| Forests | 12.5 | - | | 0.50 | 0.10* | | **0.02** | 0.21** | | **<0.001** |
|  | 25 | - | | 0.82 | 0.10* | | **0.02** | - | | **0.04** |
|  | 50 | - | | 0.77 | 0.13** | | **0.003** | - | | 0.20 |
| Grassland Shrubland | 12.5 | - | | 0.70 | - | | 0.32 | 0.05* | | **<0.001** |
|  | 25 | - | | 0.47 | - | | 0.84 | 0.05. | | **0.04** |
|  | 50 | - | | 0.54 | - | | 0.75 | - | | 0.51 |
| Urban | 12.5 | - | | 0.27 | 0.10* | | **0.01** | -- | | -- |
|  | 25 | -- | | -- | 0.10* | | **0.02** | -- | | -- |
|  | 50 | -- | | -- | - | | 0.15 | -- | | -- |
| Wetlands | 12.5 | - | | 0.25 | - | | 0.70 | - | | **<0.001** |
|  | 25 | - | | 0.39 | - | | 0.29 | - | | **<0.001** |
|  | 50 | - | | 0.16 | - | | 0.69 | - | | 0.83 |
| Elevation (m) | 12.5 | - | | 0.17 | - | | 0.22 | -- | | -- |
|  | 25 | - | | 0.20 | - | | 0.47 | -- | | -- |
|  | 50 | - | | 0.24 | - | | 0.46 | -- | | -- |
| R^2^ | 12.5 | \| 0.01 \| \| --- \| \| 0.02 \| \| 0.03 \| | | | \| 0.17 \| \| --- \| \| 0.17 \| \| 0.14 \| | | | \| 0.21 \| \| --- \| \| 0.03 \| \| 0 \| | | |
|  | 25 |  |  |  |  |  |  |  |  |  |
|  | 50 |  |  |  |  |  |  |  |  |  |
| *N* |  | | 309 | | | 61 | | | 56 | |

We show the linear model coefficients of variables with a significant effect and the significance of smooth terms for all variables. We also present the proportion of the variability the model explains (Adjusted R^2^), and the number of BBS routes included in the analyses (*N*). Significant *P-*values for the smooth terms are highlighted in bold and significant *P-*values for the linear slope are represented as follows: ****P*<0.001, ***P*<0.01, **P*<0.05. Non-significant linear slopes are denoted with a single dash (-) and predictors not included in the model are denoted with a double dash (--).

### **S7 Table. GAMMs relating the rate of change of species richness with the rate of change of the environment in US Great Plains and North American Deserts (1990 – 2019).**

|  |  | Great Plains | | North American Deserts | |
| --- | --- | --- | --- | --- | --- |
| Predictor | Resolution (km) | Linear Slope | *P*-value  smooth terms | Linear Slope | *P*-value  smooth terms |
| PPT _mean_ May-July | 12.5 | - | 0.47 | - | 0.48 |
|  | 25 | - | 0.49 | - | 0.87 |
|  | 50 | - | 0.20 | - | 0.59 |
| T_max_  May-July | 12.5 | - | 0.96 | - | 0.93 |
|  | 25 | - | 0.77 | -- | -- |
|  | 50 | - | 0.91 | -- | -- |
| T_min_  May-July | 12.5 | - | 0.98 | - | 0.30 |
|  | 25 | - | 0.82 | - | 0.34 |
|  | 50 | - | 0.38 | - | 0.81 |
| Barren | 12.5 | -- | -- | - | 0.19 |
|  | 25 | -- | -- | - | 0.37 |
|  | 50 | -- | -- | - | 0.30 |
| Crops Pasture | 12.5 | -- | -- | - | 0.21 |
|  | 25 | -- | -- | - | 0.86 |
|  | 50 | -- | -- | - | 0.38 |
| Forests | 12.5 | -- | -- | - | 0.18 |
|  | 25 | - | 0.37 | - | 0.09 |
|  | 50 | - | 0.10 | - | 0.08 |
| Grassland Shrubland | 12.5 | - | 0.13 | - | 0.30 |
|  | 25 | - | 0.15 | - | 0.41 |
|  | 50 | - | 0.22 | - | 0.09 |
| Urban | 12.5 | - | 0.53 | -- | -- |
|  | 25 | - | 0.85 | -- | -- |
|  | 50 | - | 0.24 | -- | -- |
| Wetlands | 12.5 | - | 0.22 | -- | -- |
|  | 25 | - | 0.69 | -- | -- |
|  | 50 | - | 0.54 | -- | -- |
| Elevation (m) | 12.5 | - | 0.50 | - | 0.79 |
|  | 25 | - | 0.76 | - | 0.79 |
|  | 50 | - | 0.78 | - | 0.53 |
| R^2^ | 12.5 | \| 0 \| \| --- \| \| 0 \| \| 0.06 \| | | \| 0 \| \| --- \| \| 0 \| \| 0.15 \| | |
|  | 25 |  |  |  |  |
|  | 50 |  |  |  |  |
| *N* |  | 95 | | 50 | |

We show the linear model coefficients of variables with a significant effect and the significance of smooth terms for all variables. We also present the proportion of the variability the model explains (Adjusted R^2^) and the number of BBS routes included in the analyses (*N*). Significant *P-*values for the smooth terms are highlighted in bold and significant *P-*values for the linear slope are represented as follows: ****P*<0.001, ***P*<0.01, **P*<0.05. Non-significant linear slopes are denoted with a single dash (-) and predictors not included in the model are denoted with a double dash (--).

**S8 Table. Bird assemblages (i.e., BBS routes) in the North American Deserts located near mountains or forested areas, with 30% or more of forest cover when averaged across time (1990-2019).** The proportion of forest was calculated using a 25 km buffer around the starting point of the BBS routes.

| **Location** | **Longitude** | **Latitude** | **Mean proportion of forest 1990-2019 (25 km buffer)** |
| --- | --- | --- | --- |
| CA, Alpine Village | -119.8237 | 38.7755 | 0.45 |
| CO, Meeker | -107.9698 | 40.1380 | 0.51 |
| CO, De Beque | -108.8124 | 39.4003 | 0.43 |
| ID, Tetonia | -111.2006 | 43.8248 | 0.30 |
| NM, Techado | -108.4499 | 34.6384 | 0.40 |
| UT, Eberta | -111.9544 | 39.9004 | 0.32 |
| UT, East Carbon | -110.5606 | 39.5973 | 0.32 |
| UT, Blanding | -109.4037 | 37.6749 | 0.32 |
| UT, Beaver | -112.6462 | 38.2041 | 0.54 |
| UT, Hay Canyon | -112.7998 | 37.4140 | 0.61 |
| WA, Winthrop | -120.1733 | 48.5092 | 0.64 |
| WA, Twisp | -120.2118 | 48.3551 | 0.57 |
| WY, Encampment | -106.6532 | 41.2001 | 0.43 |

REFERENCES

[1] PRISM Climate Group, “Parameter-elevation Regressions on Independent Slopes Model (PRISM) Gridded Climate Data. Recent years (1981 -2020).,” Oregon State University, Feb. 04, 2014. Accessed: Mar. 22, 2021. [Online]. Available: https://prism.oregonstate.edu/recent/

[2] T. L. Sohl *et al.*, “Modeled historical land use and land cover for the conterminous United States: 1938-1992:U.S. Geological Survey data release,” 2018. [Online]. Available: https://doi.org/10.5066/F7KK99RR

[3] T. L. Sohl *et al.*, “Conterminous United States Land Cover Projections - 1992 to 2100: U.S. Geological Survey data release,” 2018. [Online]. Available: https://doi.org/10.5066/P95AK9HP

[4] N. Nakicenovic *et al.*, *IPCC Special Report Emissions Scenarios. Summary for policymakers*. Intergovernmental Panel on Climate Change, 2000. [Online]. Available: https://www.ipcc.ch/report/emissions-scenarios/
